# The association between deficiencies in paternal and maternal reflective functioning and anorexia nervosa symptomatology

**DOI:** 10.1186/s40337-023-00836-6

**Published:** 2023-07-11

**Authors:** Dor Goshen, Daniel Stein, Jenny Kurman, Dan Farbstein, Adi Enoch-Levy, Erez Aival-Naveh, Eitan Gur, Neta Yoeli, Tali Bretler, Danny Koren, Lily Rothschild-Yakar

**Affiliations:** 1 Department of Psychology, School of Psychological Sciences, University of Haifa, Haifa, Israel; 2 Sackler Faculty of Medicine, Tel Aviv University, Tel Aviv, Israel; 3 Pediatric Psychosomatic Department, Safra Children’s Hospital, Sheba Medical Center, Tel Hashomer, Israel; 4 Center for the Treatment of Eating Disorders and Obesity, Sheba Medical Center, Tel Hashomer, Israel; 5 Ziv Medical Center, Zfat, Israel; 6 The Azrieli Faculty of Medicine, Bar-Ilan University, Poriya, Israel; 7 Psychiatry Division, Rambam Medical Center, Haifa, Israel

**Keywords:** Mentalizing, Reflective functioning, Parental reflective functioning, Anorexia nervosa, Eating disorders

## Abstract

**Background:**

A large theoretical body of knowledge exists emphasizing the importance of parental mentalizing in the context of anorexia-nervosa (AN). However, the empirical support to these assumptions is still scarce. The aim of the present study was to examine whether parents of patients with AN are characterized by a lower mentalizing ability, and whether it is associated with impaired mentalizing, AN symptomatology and eating disorder (ED) related psychological traits in the daughters.

**Methods:**

Thirty-two family triads (fathers, mothers, and daughters) of female adolescent and young adult inpatients with AN were compared with thirty-three non-clinical family triads (N = 195). The mentalizing ability of all the participants was assessed using semi-structured interviews and coded using the Reflective Functioning Scale (RFS). Self-report questionnaires were administered to the daughters to evaluate ED symptomatology and ED related psychological traits (e.g., low self-esteem, interpersonal insecurity, emotional dysregulation).

**Results:**

Decreased reflective functioning (RF) levels were found among mothers and fathers of patients with AN compared to their control peers. Examining the entire sample, clinical and non-clinical groups together, showed that both paternal and maternal RF were associated with the daughters' RF and each were found to have a significant and distinct contribution to the daughters' RF. Significant associations were found between lower levels of maternal and paternal RF and increased ED symptoms and ED related psychological traits. The use of a mediation model suggested a serial relationship in which low maternal and paternal RF contributes to the daughters' low RF, which in turn is associated with higher levels of psychological maladjustment, and ultimately contributes to the increased severity of ED symptoms.

**Conclusions:**

The present results provide strong empirical support for theoretical models that suggest that deficits in parental mentalizing may represent important correlates of the presence and severity of ED symptoms in AN. Furthermore, the results highlight the relevance of fathers' mentalizing ability in the context of AN. Finally, clinical and research implications are discussed.

## Abstract (plain language summary)

The goal of the study was to examine the relationship between deficits in parents' ability to reflect on their own and others' emotional experience and the severity of their daughter's eating disorder and psychological maladjustment. A clinical group of young female inpatients diagnosed with anorexia-nervosa and both their biological parents was compared to a control group of young females without eating disorder and their parents. Personal interviews assessed the participants' reflective ability and self-report questionnaires assessed the daughters' eating disorder severity and psychological difficulties. The results show that patients with anorexia nervosa and their parents are less able to reflect on their emotional experiences compared to the control group. The parents' reflective ability was found to be related to their daughter's reflective ability. For both fathers and mothers, the less able the parent is to reflect on his or her emotional experiences the higher the severity of the daughter's eating disorder symptoms and psychological maladjustment are. The results imply that deficits in the parents' ability to process their own and others' emotional experiences are related to the severity of eating disorders symptoms and psychological maladjustment among daughters.. The study results highlight the importance of assessing reflective abilities of fathers.

## Background

Anorexia nervosa (AN) is considered one of the most severe mental illnesses due to high rates of overall morbidity and functional decline [1]. The onset of AN occurs most commonly during early adolescence and is characterized by extreme dieting aimed at weight loss, obsessive preoccupation with body weight and shape, and a strive to control it [2]. The etiology of AN is complex and includes a combination of genetic, developmental, psychological, familial, and socio-cultural factors [3]. Similarly, the treatment of AN is challenging, and while success rates have increased with the development of illness-specific approaches, optimization of treatment is still required [4].

In recent years, an expanding body of knowledge has been conceptualizing AN in light of the *mentalization model*. The construct of *mentalizing* refers to the human capacity to reflect and interpret one's own and others' behavior in terms of internal mental states such as thoughts, feelings, and intentions [5]. A core assumption in this model is that mentalizing is not a single capacity, but rather as a set of related and partially overlapping psychological abilities, such as *empathy*, *alexithymia*, and *theory of mind (ToM)* [6]. Mentalizing capacities begin to develop during infancy and childhood and are heavily dependent on the relationship between the child and his or her primary attachment figures [7]. From this vantage point, mentalizing is considered a developmental achievement that is necessary in order to develop adequate coping mechanisms for dealing with stress, affect regulation skills, and the formation of stable interpersonal relationships [8].

The concept of mentalizing has been translated into an operational and empirically measurable one termed *Reflective Functioning* (RF; [5]). This concept refers to the reflective abilities of the individual that allows one to distinguish between external and internal reality, fictitious and actual reality, and to distinguish emotional and intrapersonal processes from interpersonal communication. A high RF level indicates a person's ability to acknowledge that perceived mental states are subjective, and therefore may be misguided or change with time [9].

Parental mentalizing, or parental RF (PRF), refers to the ability of parents and other caregivers to "keep the child's mind in mind", by seeing the child as a psychological entity and interpreting his or her behavior in terms of mental states [10]. A large body of knowledge emphasizes the positive effect of well-developed PRF on parenthood and, in turn, on the child's socio-emotional development [11]. Elevated PRF levels were found to be associated with more adequate and sensitive parenting [12–14], an increased likelihood of transmitting secure attachment [15, 16] and the promotion of self-regulation in children [17]. In contrast, deficits in PRF were found to be associated with less favorable results, such as difficulties in parents' ability to regulate their toddler's distress [18] and higher rates of externalizing behaviors among children [19]. Additionally, studies have demonstrated intergenerational transmission of mentalizing abilities from parent to child and found a positive correlation between PRF and the child's RF [20, 21]. However, almost all the studies conducted on the effect of PRF on children's psychopathology and well-being have focused on mothers and their infants or school-aged children, making it difficult to deduce the effect of PRF on later stages of development, such as adolescence and adulthood [22].

The role of paternal RF in children's development has been exemplified in only a handful of studies. These studies found that increased paternal RF is associated with secure attachment in children [23], an increased likelihood of emotional engagement and supportive behavior during interactions of fathers with their infants [24], lesser behavioral disturbances, and a more stable perception of self and others during adolescence [25]. In another study, both maternal and paternal RF were found to correlate with adolescents' RF, but only paternal RF was also associated with adolescent's sense of social competence [26]. These findings highlight the need to expand the body of knowledge pertaining to the role of paternal RF in children and adolescents' development and psychopathology.

### Mentalizing in anorexia nervosa

The last two decades have seen a growing body of knowledge conceptualizing the development and maintenance of AN in light of *the mentalization model* [27]. Skårderud and Fonagy [28] view AN as a failure to establish a differentiation between bodily sensations and emotional states and between one's own and others' emotional experiences. Lacking a cohesive sense of self leads patients with AN to act as if their body has a central role in preserving the continuous experience of the self. In this sense, ED symptoms are conceptualized as an attempt to establish a sense of viability, and self-regulation [28, 29]. In line with this theoretical model, empirical studies show that in comparison to non-clinical samples, patients with AN are characterized by lower RF levels [30, 31], a higher prevalence of alexithymia [32], and difficulties in recognizing others' emotions [31, 33]. However, these studies are cross-sectional and causality is yet to be established.

In the context of EDs, parental mentalizing is thought to play a pivotal role. The developmental mentalization model suggests that deficits in the parent's ability to ponder about his or her child's emotional experience and then adequately reflect the emotional experience back to the child, may result in the child's inability to comprehend his or her inner world, putatively leading to the development of ED symptoms as a concrete means of emotional regulation [28]. However, despite the vast body of research demonstrating reduced mentalizing abilities in adolescent and adult patients with AN [31–34], only a handful of studies have examined parents' mentalizing abilities in the context of EDs. Specifically, there is no data on the RF of fathers of patients with EDs and only one study has examined the RF levels of mothers of patients with AN [35]. This study was conducted on a limited number of mothers (*n* = 12), and found deficits in maternal RF. In a recent study conducted by Jewell et al. [36] excessive certainty among parents regarding their own mental states, which is characteristic of low mentalizing ability, as measured by a self-report RF questionnaire (RFQ8; [37]), predicted negative treatment outcome in AN-focused family therapy.

The few studies that have examined similar concepts such as alexithymia or ToM among parents of patients with EDs provided conflicting results. While some studies demonstrated an increased prevalence of alexithymia in parents of patients with AN in comparison to parents in a non-clinical sample [38, 39], other studies did not find such differences [40–42]. Regarding ToM, to the best of our knowledge, only one study compared emotion recognition in mother-daughter dyads in samples of patients with binge/purge type EDs and healthy controls (HCs). In this study, no between-group differences were found in overall maternal emotion recognition, but mothers of patients with EDs revealed an increased tendency to ignore negative emotions compared to mothers of HCs [42].

In summary, there appears to be a significant gap between the central role attributed to parental mentalizing in theoretical models and the paucity of data from empirical studies to support these models. Particularly salient is the lack of empirical data regarding paternal mentalizing in the context of AN as well as the lack of studies comparing both fathers and mothers of ED patients to fathers and mothers of HCs. Furthermore, to the best of our knowledge, no previous study has examined the relationship between parents' mentalizing ability and the severity of ED symptoms among their children, making it difficult to establish the role of parental mentalization in the development and maintenance of EDs.

### Aims and hypotheses

The overarching goal of the current study was to examine the associations between maternal and paternal mentalizing ability and the presence and severity of AN symptomatology and ED related psychological traits among daughters.

The study hypotheses were as follows:

1. The RF levels of patients with AN would be lower than that of their HC counterparts.
2. The RF levels of mothers and fathers of patients with AN would be lower than that of HC parents.
3. Maternal and paternal RF levels would positively relate to the daughters' RF level.
4. Daughters' and parents' RF levels would be negatively associated with the daughters' severity of ED and ED related psychological traits. As an open question we sought to examine whether maternal and paternal RF contribute differently to the severity of the ED and ED related psychological traits.
5. The daughters' RF and psychological maladjustment would serve as mediating factors in the association between the parents' RF and the severity of the daughters' ED symptoms.

## Methods

### Overall design

To test the above hypotheses, the study compared a clinical sample of female adolescent and young adult inpatients and both of their parents with a non-clinical control sample of daughters and parents from the general population.

### Participants

Participants included 65 female adolescents and young adults between the ages of 14–24 (*M* = 17.00, *SD* = 2.23) and both of their biological parents (*N* = 195).

*The clinical group (AN)* included 32 triads of female inpatients with AN and both of their biological parents. Patients were hospitalized in inpatient departments at two medical centers in Israel: Sheba Medical Center at Tel Hashomer, or in the Ziv Medical Center in Safed, and in adult ED inpatient department at the Sheba Medical Center.. All patients met the DSM-5 [43] criteria for diagnosis of AN at admission.

Inclusion criteria were female gender, between 14–24 years of age, having a good understanding of the Hebrew language, and consent of the patients and their parents to take part in the study.

Exclusion criteria were having a lifetime or current diagnosis of DSM-5 [43] bipolar disorder, schizophrenia spectrum disorder, substance use disorder, intellectual disability, organic brain syndrome, and any physical disorder other than intercurrent medical problems. Patients were diagnosed with either AN-restricting type (AN-R; *n* = 21) or AN binge/purge type (AN-B/P; *n* = 11). Sixteen patients (50%) were diagnosed with comorbid depressive disorders, five (16%) with comorbid anxiety disorders, and one (3%) with obsessive compulsive disorder (OCD). Two participants were excluded from the study because one of their parents had dropped from the study.

*The HC group* included 33 triads of healthy female adolescents and young adults, and both of their biological parents. HC daughters were required to have no lifetime or current history of any psychiatric illness, no chronic physical illness requiring continuous medication or frequent hospitalization, and no signs or symptoms indicative of an ED.

All participants gave their written informed consent to participate in the study after receiving an explanation regarding the study's goals and methodology. The study was approved by the Ethical Committee of Haifa University, Israel (Protocol No.169/16; June 17^th^, 2016), the Internal Review Boards (Helsinki Boards) of Sheba Medical Center, Tel Hashomer, Israel (Protocol No. 2755; January 28^th^, 2016), and Ziv Medical Center, Zefat, Israel (Protocol No. 1417; February 14^th^, 2016).

The sample size (*N* = 195) was determined based on an a priori power analysis that assumed a power of 80%, α = 0.05 and medium effect size, consistent with previous studies on the mentalizing abilities of patients with AN and controls [30] and studies about the associations between parents' and adolescents' RF levels [26].

Participants in the HC group were recruited from the general population using snowball sampling, and were matched to the clinical group on age, parental education, parental marital status, and estimated IQ as assessed using two subtests (Block Design and Similarities) from the Hebrew edition of the Wechsler Adult Intelligence Scale (WAIS-III^Heb^, [44]; WISC-IV^Heb^, [45]).

### Measures

#### Diagnosis of AN and comorbid psychiatric disorders

Diagnosis of AN and of comorbid psychiatric disorders was obtained using a semi-structured interview, based on the principles of the *Structured Clinical Interview for DSM-IV Axis I Disorders, Patient Edition, Version 2.0*; *SCID-I/P Version 2.0* [46], and adapted to the DSM-5 [43] diagnostic criteria.

#### The SCOFF-questionnaire

Morgan et al. [47] includes 5—yes/no questions assessing the presence of ED symptoms in community populations. A total score of 2/5 and above has been found to be 100% sensitive and 87.5% specific for the presence of disordered eating [47]. HC participants answering positively to any of the SCOFF questions were excluded from the study.

#### The Reflective Function Scale (RFS; [5])

To measure the parents and daughters' mentalizing ability we used the RF coding system on the Adult Attachment Interview (AAI-RF; [5]). The AAI [48] is a semi-structured interview in which interviewees are requested to describe their relationships with their parents during childhood, addressing issues such as closeness, experiences of rejection and loss, and the way in which their overall childhood experience influenced the formulation of their personality. AAI verbatim transcripts were encoded into a numerical RF score, representing the measured willingness and capacity of the interviewees to observe mental states coherently. The RFS is a six-level scale, ranging in odd numbers from (− 1), which represents an oppositional stance, distorted, bizarre, or inappropriate mentalizing, to 9, which represents exceptionally complex, original or sophisticated mentalizing.

The Hebrew translation of the AAI was validated in a previous study [49]. In the current study, all interviews were recorded using a sound-recorder and transcribed by research assistants. Two of the authors (D.G. and L.R.Y.) coded the RF-AAI protocols, both having passed the Anna Freud Centre's reliability test with good results. The coders were not exposed to the participants' name or any of their task/questionnaire details or results. Reliability rating was performed on ten interviews in each subgroup (overall 60 protocols), yielding high inter-rater reliability, with an intraclass correlation coefficient (ICC) = 0.86 for RF scores.

#### The Eating Attitudes Test (EAT-26; [50])

The EAT-26 is a self-report questionnaire that examines the level of pathological eating-related preoccupations and behaviors. Participants rate 26 items on a six-point scale ranging from never (1) to always (6), with higher scores indicating greater disturbance. A score of ≥ 20 indicates likelihood of disordered eating. The Hebrew translation of the EAT-26 has previously been shown to successfully differentiate Israeli patients with EDs from non-ED controls [51]. In the current study, the questionnaire was administered to daughters from the AN and HC groups. Cronbach's alpha of the EAT-26 in this study = 0.97.

#### The Eating Disorder Inventory-3 (EDI-3; [52])

To measure ED related psychological traits we used the EDI-3. It is a self-reported questionnaire comprising 91 items answered on a six-point Likert scale. The EDI-3 is composed of 12 subscales—three measure ED symptom (drive for thinness, bulimia, and body dissatisfaction) and 9 measure psychological traits characterizing ED pathology low self-esteem, personal alienation, interpersonal insecurity, interpersonal alienation, introspective deficits, emotional dysregulation, perfectionism, asceticism, and maturity fears). The subscales yield five composites scores: eating disorder risk (EDRC), ineffectiveness (IC), interpersonal problems (IPC), affective problems (APC), and overcontrol (OC). Additionally, the EDI-3 provides a general psychological maladjustment composite (GPMC) which is composed of the four psychological subscales (IC, IPC, APC, and OC). Studies that assessed the psychometric qualities of the EDI-3 found excellent reliability and structure validity among clinical samples and good reliability and structure validity among non-clinical samples [52, 53]. Among the non-clinical sample, the Asceticism scale was repeatedly found to have borderline level of internal consistency [54–56].

The Hebrew translation of the EDI-2, which includes the same items as the EDI-3 but is scored differently, has previously been shown to successfully differentiate Israeli patients with EDs from non-ED controls [57]. In the current study, the questionnaire was administered to the daughters from the AN and HC groups and we used only the composites that describe psychological traits characterizing ED pathology (IC, IPC, APC, OC, and GPMC), which all revealed good internal consistencies in this study: IC: α = 0.95, IPC: α = 0.93, APC: α = 0.91, OC: α = 0.84, and GPMC: α = 0.97.

### Procedure

Patients with AN were interviewed on admission with the SCID-I/P Version 2.0 by certified psychiatrist (E.G.) and child and adolescent psychiatrists (A.E.L., D.S., & T.B.), all of whom are highly experienced. Diagnoses were confirmed in clinical team meetings of the respective departments. Controls were interviewed using the SCID-I/P Version 2.0 and the SCOFF screening items by masters level psychology students trained for using these tools by a senior psychiatrist (D.S.). Only participants with negative answers on all the SCID-I/P Version 2.0 screening items and the SCOFF items were included in the HC group. One control HC participant was excluded from the study as she did not fulfilling the study's criteria. This method of identifying HC participants has been used previously in ED studies [58], including in Israeli samples [59].

The study measures were administered by Ph.D. and M.A clinical psychology students. The study task was administered individually to each participant, apart from their other family members.. Sessions with patients and parents from the AN group took place in the respective inpatient departments. Patients with AN and their parents were assessed within two weeks of admission after their overall medical condition had stabilized, as determined by physical examinations and relevant laboratory tests. This was done to reduce the influence of the patients' physical condition on the study findings. The sessions with participants from the HC group took place in their homes, in a separate room and without the presence of any other family members.

### Data analyses

Analyses were conducted using SPSS version 28.0 with significance put as p < .05. The degree of matching between the AN and HC groups was tested using a series of t-tests for continuous variables and chi-square tests for dichotomous variables. To examine differences in the dependent variables we conducted multivariate analyses of variance (MANOVAs) models using Hotelling's Trace criterion, with group membership (AN versus HC) as the independent variable, and daughters' ED symptoms (EAT-26) and the five psychological composites of the EDI-3 as the dependent variables. The first and the second hypotheses, regarding the differences in RF levels between the AN and HC groups were tested using a two-factor mixed between-within MANOVA with clinical group (AN versus HC) as the between-subjects factor and family role (daughter, mother, father) as the within-subjects factor, and RF as the dependent variable. To explore the source of the differences in the within-subjects factor, we used two simple contrasts of mothers and fathers versus daughters. Analyses of the third, fourth, and fifth hypotheses were carried out on the entire sample. The third hypothesis, concerning the associations between parents and daughters' RF was tested using partial Pearson correlation coefficients and linear regression analyses, with paternal and maternal RF as the predictors, and the daughters' RF as the dependent variable. The fourth hypothesis, regarding the associations between the participants' RF levels and the daughters' ED symptoms and ED related psychological traits were tested using partial Pearson correlation coefficients, and a series of five hierarchical linear regression analyses.

For the fifth hypothesis, regarding the potential mediating role of the participants' RF and psychological maladjustment in the association between parental RF and ED severity we applied mediation analyses. The analyses were conducted according to Hayes and Preachers' guidelines [60] using the PROCESS macro for SPSS (v.3.3; [61]). This application allows for the testing of multiple paths while employing a bootstrapping procedure with 5000 resamples.

## Results

Table 1 compares the demographic characteristics and background variables of the AN and the HC groups using independent t-tests for continuous variables and chi-square test for dichotomous variables (e.g., marital status of parents). The data revealed no significant between-group differences for age, estimated intelligence quotient (IQ) measures, parental education, and marital status. Patients with AN reported significantly lower body mass index (BMI) compared to the HC participants.

**Table 1:** Between-group differences in demographic variables, estimated IQ and BMI

	AN (*n* = 32)	HC (*n* = 33)	*F*(1, 64)	*P*
	*Mean (SD)*	*Mean (SD)*		
*Daughters*				
Age	17.54 (2.34)	16.77 (2.06)	1.55	.18
Education	10.81 (1.31)	10.69 (1.24)	.13	.72
IQ-similarities	10.35 (2.75)	10.84 (2.17)	.62	.38
IQ-block design	10.52 (3.25)	11.22 (2.66)	.88	.35
daughters' BMI	17.92 (1.83)	20.76 (2.64)	25.24	.0001
*Mothers*				
Age	48.47 (5.53)	48.88 (5.53)	.11	.75
Education	14.81 (2.40)	15.81 (1.99)	3.39	.07
IQ-similarities	10.53 (2.11)	11.18 (1.96)	1.66	.20
IQ-block design	10.59 (2.96)	11.27 (2.23)	.78	.38
*Fathers*				
Age	51.03 (5.10)	51.24 (6.20)	.02	.88
Education	15.00 (2.79)	15.72 (2.13)	1.40	.24
IQ-similarities	11.48 (2.00)	11.09 (2.28)	1.28	.27
IQ-block design	11.26 (3.00)	12.12 (2.45)	1.60	.20
*Parents' marital status*			χ^2^ = 1.37	.50
Married	93.8%	90.9%		
Divorced	6.2%	6.1%		
Single	.00%	.00%		

*AN* anorexia-nervosa, *HC* healthy control, *IQ* intelligence quotient, *BMI* body mass index

### Between-group differences in ED symptomatology and ED related psychological traits

First, we sought to examine between-group differences in the dependent variables of the study, using a one-way MANOVA. The results indicated a significant and strong effect of group membership (*Hotelling T* = 3.47, *F*_(6,58)_ = 33.54, *p* < 0.0001, *η*^*2*^ = 0.78). Patients with AN reported on significantly higher levels of ED symptoms as well as higher levels of ineffectiveness, interpersonal problems, affective problems, over-controlled behaviors, and general psychological maladjustment compared to HC participants (see Table 2).

**Table 2:** Between-group differences in ED symptoms and ED related psychological traits

Variables	*AN (N* = *32)*		*HC (N* = *33)*		*F*	*η*^*2*^
	*M*	*SD*	*M*	*SD*		
EAT-26	50.81	17.62	8.70	7.01	162.18***	.72
*EDI-3*						
IC	55.66	10.42	36.91	6.56	75.19***	.54
IPC	54.31	11.32	38.39	8.80	47.58***	.43
APC	54.84	11.82	42	11.71	27.71***	.31
OC	52.63	8.66	42.36	6.30	29.96***	.32
GPMC	55.00	9.87	39.18	6.52	58.48***	.48

*EAT-26* Eating Attitude Test 26, *EDI-3* Eating Disorder Inventory 3, *IC* Ineffectiveness Composite, *IPC* Interpersonal Problems Composite, *APC* Affective Problems Composite, *OC* Overcontrol composite, *GPMC* general psychological maladjustment

***p < .001

### Between-group differences in RF levels

**Fig. 1:**
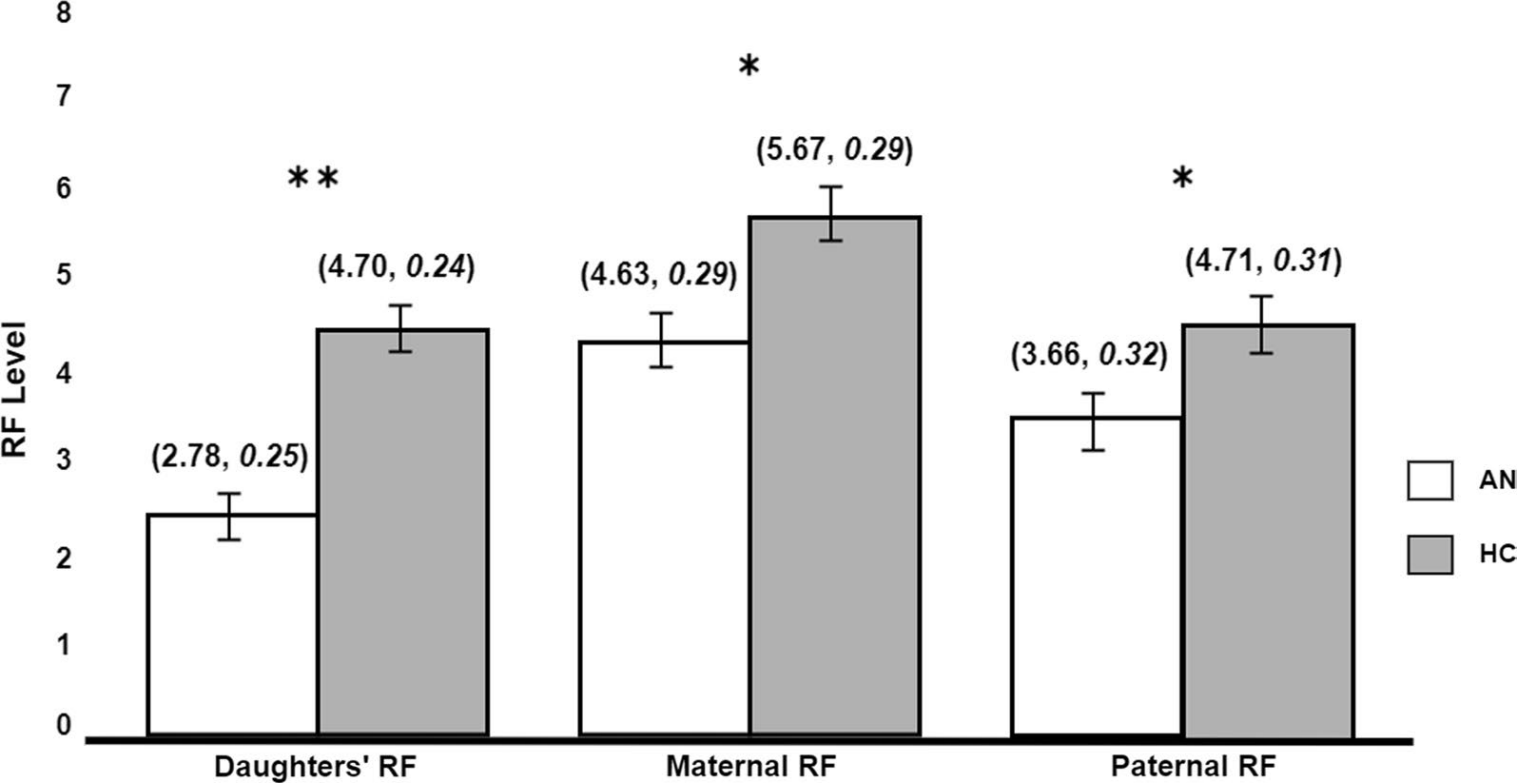
Between group differences in RF levels. *p < .05; **p < .001 (mean, standard error). *RF* reflective-functioning. *AN *anorexia nervosa. *HC *healthy controls

Figure 1 presents the means of RF levels in the two groups by family roles. A two-way mixed between-within MANOVA with group membership as a between-subjects factor and family role (daughter, mother, and father) as the within-subjects factor was performed. In line with our first and second hypotheses, the results indicated a significant group effect (AN vs. HC) with a small to medium effect size (*Hotelling T* = 0.49, *F*_(3,61)_ = 10.05, *p* < 0.0001, *η*^*2*^ = 0.33). A series of one-way ANOVAs revealed that RF scores of daughters, mothers and fathers from the AN group were significantly lower in comparison to their HC peers (*F*_(1,63)_ = 30.68, *p* < 0.0001, *η*^*2*^ = 0.33; *F*_(1,63)_ = 6.58, *p* < 0.05, *η*^*2*^ = 0.10; *F*_(1,63)_ = 6.547, *p* < 0.05, *η*^*2*^ = 0.09; respectively). Next, the within factor showed a significant effect for the family role (*F*_(1,63)_ = 21.95, *p* < 0.0001, *η*^*2*^ = 0.26) and a marginally significant effect for the family role and group membership interaction (*F*_(1,63)_ = 2.48, *p* = 0.09, *η*^*2*^ = 0.04). A further simple contrasts analysis pointed to a significant group X family role interaction effect in the comparison between mothers and daughters, demonstrating that the difference between the daughters and the mothers' RF is more prominent in the AN group compared to the HC group, with daughters having lower mentalizing abilities compared to mothers (*F*_(1,63)_ = 4.13, *p* < 0.05, *η*^*2*^ = 0.06).

### The associations between parental and daughters' RFs

In accordance with our third hypothesis, significant positive correlations were found between maternal and paternal RF and daughter's RF (see Table 3). Additionally maternal and paternal RF were also positively correlated. To test the joint contribution of paternal and maternal RF, a regression model was computed. The regression model was significant (*F*_(2,62)_ = 11.52, *p* < 0.001) and explained 25% of the variance in the daughters' RF. Both maternal RF (*β* = 0.27, *p* < 0.05) and paternal RF (*β* = 0.31, *p* < 0.05) remained positive explaining factors of daughters' RF. This result indicated that despite the correlation between them, maternal and paternal RF each had a unique contribution to the explanation of their daughter's RF.

**Table 3:** Pearson correlation coefficients between parents and daughters' RF, ED symptoms, and ED related psychological traits (N = 65)

	D-RF	M-RF	P-RF
M-RF	.45***	1	
P-RF	.47***	.58***	1
EAT-26	− .43***	− .28*	− .27*
*EDI-3*			
IC	− .50***	− .37**	− .36**
IPC	− .46***	− .31*	− .36**
APC	− .33**	− .40**	− .36**
OC	− .15	− .13	− .25*
GPMC	− .41***	− .36**	− .36**

*D-RF* daughters' Reflective-Functioning, *M-RF* maternal Reflective-Functioning, *P-RF* paternal Reflective-Functioning, *EAT-26* Eating Attitude Test 26, *EDI-3* Eating Disorder Inventory 3, *IC* Ineffectiveness Composite, *IPC* Interpersonal Problems Composite, *APC* Affective Problems Composite, *OC* Overcontrol composite, *GPMC* general psychological maladjustment

*p < .05; **p < .001; ***p < .0001

### The associations of parents' and daughters' RF with ED symptoms and ED related psychological traits

The fourth aim of the study was to explore whether lower levels of daughters and parents' RF are associated with ED symptoms and ED related psychological traits. First, correlation coefficients were computed between parents and daughters' RF scores and the variables assessing ED severity and ED related psychological traits (see Table 3). In line with our hypothesis, significant negative correlations were found between the daughters, mothers, and fathers' RF and the severity of ED symptoms, i.e., lower RF was associated with greater ED symptomatology. Among the EDI-3 variables, significant negative correlations were found between the daughters, mothers, and fathers' RF and the following composites: IC, IPC, APC, and GPMC. The OC score negatively correlated with fathers' RF, but not with the mothers or daughters' RF.

Next, we conducted a series of hierarchical regression analyses across the study groups to examine the ability of the daughters and the parents' RF to explain the findings regarding ED symptoms and ED related psychological traits. In each model, maternal and paternal RF were entered in the first step and the daughters' RF was added in the second step. The results of the regression analyses are presented in Table 4. In the explanation of ED severity, the regression model was significant (*F*_(3,61)_ = 4.89, *p* < 0.001) explaining 15% of the variance. The first step was significant and explained 7% of the variance in the daughters' ED symptoms (*F*_(2,62)_ = 3.32, *p* < 0.05). It is of note that unlike the simple correlations, none of the predictors—maternal and paternal RF—was a significant explaining factor, likely due to their common variance. In the second step, the addition of the daughters' RF was significant and contributed 10% to the explained variance (*F*_(1,61)_ = 7.37, *p* < 0.05). In this step, the daughters' RF score was the only significant negative explaining factor of ED severity.

**Table 4:** Summary of hierarchical regression models explaining ED severity and ED related psychological symptoms by parental RF with and without daughters' RF

Predictors	EAT-26		IC		IPC		APC		OC	
	β	R^2^_change_	β	R^2^_change_	β	R^2^_change_	β	R^2^_change_	β	R^2^_change_
*Step 1*		10*		.17*		.17*		.18*		.06
M-RF	− .18		− .25^†^		− .14		− .29*		.02	
P-RF	− .17		− .16		− .31*		− .19		− .26^†^	
*Step 2*		10*		.11*		.09*		.02		.00
M-RF	− .08		− .14		− .05		− .25		.04	
P-RF	− .05		− .09		− .20		− .15		− .25	
D-RF	− .37**		− .39*		− .34*		− .15		− .05	
ΔR^2^		.15*		.24***		.22**		.16*		.02

*D-RF* daughters' Reflective-Functioning, *M-RF* maternal Reflective-Functioning, *P-RF* paternal Reflective-Functioning, *EAT-26* Eating Attitude Test 26, *EDI-3* Eating Disorder Inventory 3, *IC* Ineffectiveness Composite, *IPC* Interpersonal Problems Composite, *APC* Affective Problems Composite, *OC* Overcontrol Composite

^†^p = .08; *p < .05; **p < .001

^a^R^2^ in step 1, ^b^R^2^ in step 2

The model explaining the daughters' IC was significant (*F*_(3,61)_ = 7.88, *p* < 0.0001) explaining 24% of the variance. The first step of the regression was significant explaining 14% of the variance (*F*_(2,62)_ = 6.35, *p* < 0.05). In this step maternal RF was a marginally significant explaining factor. In the second step, the addition of the daughters' RF was significant and added 11% to the explained variance (*F*_(1,61)_ = 9.23, *p* < 0.05). In this step, the daughters' RF was the sole significant explaining factor for the IC score.

The model explaining the daughters' IPC was significant (*F*_(3,61)_ = 6.45, *p* < 0.001) explaining 20% of the variance. The first step of the regression was significant and explained 12% of the variance (*F*_(2,62)_ = 5.43, *p* < 0.05). In this step, the paternal, but not maternal RF score was found to be a significant negative explaining factor. In the second step, the addition of the daughters' RF was significant and added 9% to the explained variance (*F*_(1,61)_ = 7.39, *p* < 0.05). As in the previous analyses, in this step the daughters' RF was the only significant explaining factor, while paternal RF was no longer significant explaining factor.

The model explaining the daughters' APC was significant (*F*_(3,61)_ = 5.10, *p* < 0.05) explaining 16% of the variance. The first step of the regression was significant explaining 18% of the variance (*F*_(2,62)_ = 6.97, *p* < 0.05). In this step, the maternal, but not paternal RF score was found to be a significant negative explaining variable. As opposed to the patterns that were found in the regression analyses of the other composites scores, here the addition of the daughters' RF in the second step did not have a significant contribution for the explained variance (*F*_(1,61)_ = 1.29, *p* = 0.26).

Last, the regression model for explaining adolescent OC was not significant (*F*_(3,61)_ = 1.41, *p* = 0.25).

### The mediating role of daughters' RF and psychological maladjustment in the association between parental RF and ED severity

**Fig. 2:**
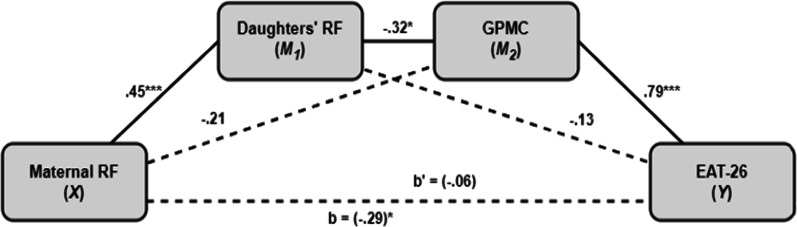
Mediation model of the associations between maternal RF and ED severity. *p < .05; ***p < .0001. *RF* reflective functioning, *GPMC* general psychological maladjustment composite

**Fig. 3:**
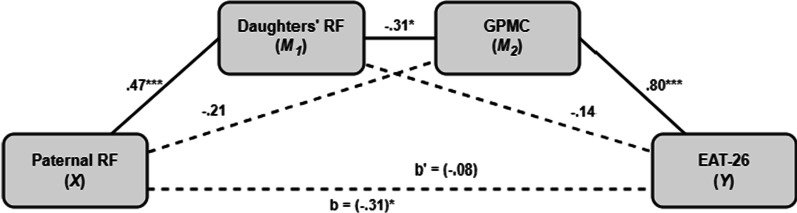
Mediation model of the associations between paternal RF and ED severity. *p < .05; ***p < .0001. *RF* reflective functioning, *GPMC* general psychological maladjustment composite

Following the fifth hypothesis, we examined possible mediators in the relationship between parental RF and the severity of ED symptoms. To this end, we employed two parallel multiple mediation models with parental (maternal/paternal) RF as an independent variable, daughters' ED symptoms (EAT-26 score) as the dependent variable and two potential mediators in the following order: daughters' RF, and GPMC. The results are presented in Figs. 2 and 3.

In both models, the results indicated a serial mediation path with the daughters' RF and GPMC serving as significant mediators (X → M_1_ → M_2_ → Y) between maternal/paternal RF and the severity of ED symptoms (*effect* =  − 0.34, *CI*_*bootstrap*_: − 0.53 to − 0.14; *effect* =  − 0.35, *CI*_*bootstrap*_: − 0.53 to − 0.18; prospectively). In these models, elevated maternal and paternal RF levels decreased the severity of the daughters' ED symptoms through a positive direct effect on the daughters' RF (*effect* = 0.45, *SE* = 0.11, *t*_(65)_ = 4.03, *p* < 0.0002; *effect* = 0.47, *SE* = 0.10, *t*_(65)_ = 4.22, *p* < 0.0001; prospectively), subsequent negative direct effect of the daughters' RF on GPMC score (*effect* =  − 0.32, *SE* = 0.86, *t*_(65)_ =  − 2.48, *p* < 0.05; *effect* =  − 0.31, *SE* = 0.87, *t*_(65)_ =  − 2.43, *p* < 0.05; prospectively), and finally, a positive direct effect of the GPMC score on the severity of ED symptoms (*effect* = 0.79, *SE* = 0.17, *t*_(65)_ = 9.67, *p* < 0.0001; *effect* = 0.80, *SE* = 0.17, *t*_(65)_ = 10.03, *p* < 0.0001). In both models, there was no evidence for a direct effect of maternal or paternal RF on daughters' ED symptoms (*effect* = 0.06, *SE* = 1.19, t_(65)_ = 0.77, *p* = 0.44; *effect* = 0.08, *SE* = 1.10, t_(65)_ = 0.94, *p* = 0.35), indicating that the association between the parents' RF and the daughters' ED severity is fully mediated by the daughters' RF and GPMC score.

## Discussion

The overarching goal of the current study was to investigate the relationship between paternal and maternal RF and the presence and severity of ED symptoms and ED related psychological traits among daughters. In addition, the study focused on assessing paternal RF, a field that had not previously been examined empirically in the context of ED. Indeed, the results of this pioneering study provide initial empirical evidence indicating a relationship between lower levels of maternal and paternal RF and a higher prevalence of ED symptoms and psychological maladjustment among daughters.

### Deficient mentalizing ability in female patients with AN and their parents

In line with our first and second hypotheses, the study findings reveal that fathers, mothers, and daughters with AN are characterized by lower RF levels compared to their HC peers. These findings support the hypothesis that deficits in mentalizing characterize not only the AN patients themselves but also their parents [28].

The lower RF levels found among mothers from of the AN group are congruent with the findings of the single study that investigated the matter [35]. As far as we know, the current study is the first to find that fathers of daughters with AN are characterized by lower RF levels compared to fathers of HC daughters. Interestingly, while maternal RF levels among the AN group were found to be significantly lower compared to the HC mothers, they were only slightly lower than average RF levels among the general population, corresponding to the lower range of the “ordinary RF” category [5]. This indicates that mothers of patients with AN have the potential to use their mentalizing abilities yet in a more limited and undeveloped way. On the other hand, in the AN group, the average paternal RF level was in the “questionable or low RF” range. This category includes the use of pre-mentalizing patterns characterized by pseudo-mentalizing, the overuse of clichés, and referring to emotional states in a banal manner [5]. In fact, the only subgroup scoring lower than fathers of patients with AN were the patients themselves. In accordance with previous findings, in our study AN patients demonstrated deficient RF levels that were indicative of concrete thinking patterns, with little reference made to mental states [30, 62].

These results highlight the critical need to cast a spotlight on the mentalizing ability of fathers of patients with AN, who demonstrated more significant deficiencies compared to their HC peers, as opposed to mothers from the AN group, who also demonstrated decreased RF levels, yet as aforementioned, in a more moderate way.

From a different perspective, the interaction effect that was found in the contrast analysis demonstrates that the difference between the mentalizing abilities of daughters and mothers is more prominent in the AN group compared to the HC group, with daughters having lower mentalizing abilities compared to mothers. These results suggest that the family role (e.g., mother/daughter) may not be the only factor that explains the lower mentalizing ability found in AN patients but that the AN related characteristics (e.g., concrete thinking) may be an additional contributing factor.

### The association between parental and daughters' RF

The importance of addressing the mentalizing abilities of both parents, lies in the positive relationships found in our study between the RF levels of both fathers and mothers and that of their daughters. It appears the parents' ability to reflect and ponder about their own emotional experiences within their attachment relationships contributes significantly to their adolescent child's ability to do so.

Beyond contributing to the body of knowledge on EDs, our findings may be important also in a broader context. First, most studies examining the relationship between parents and children's mentalizing abilities have been conducted on samples of mothers and their infants or children typically aged 6–13 [18, 63, 64]. Our findings suggest that there might be a meaningful relationship between parents and daughters mentalizing abilities, in female adolescents and young adults, both healthy and with AN. This finding supports the notion that parental mentalizing abilities continue to be relevant during adolescence and young adulthood [65]. This is further reinforced by neuroscience research showing that areas of the brain related to social cognition and self-awareness continue to develop throughout adolescence [66] and that parents and the close environment in general may play a major role in creating the conditions for the continued development of those abilities [67].

On another level, the regression analysis results indicate that while the RF of both parents contribute to the daughters' RF, paternal RF has a slightly higher contribution than maternal RF. Similarly, Benbassat and Priel [26] found the RF levels of community male and female adolescents to be significantly related to the RF levels of both of their parents, and they too observed a stronger relationship to paternal RF. In an attempt to explain this, Benbassat and Priel argued that the father's influence becomes more significant during adolescence, as he is the one who enables the second phase of his child's separation-individuation process, which helps the adolescent gain independence [22, 68]. While contemporary theories of family roles challenge traditional dichotomous views of stereotypic paternal and maternal roles [69], it seems that the contribution of fathers to their children's mental and emotional development often tends to be easily neglected due to the sweeping tendency to observe and focus on maternal variables. For example, a longitudinal study [70] examining the ability of maternal and paternal attachment patterns assessed during pregnancy to predict the RF of children at the age of 16 found that while maternal attachment patterns predicted the adolescents' RF levels, paternal attachment patterns did not. As a result, the researchers cautiously raised the question of whether fathers may be irrelevant to their children's emotional development [70]. In contrast, our findings suggest that there might be a strong relationship between the fathers' and daughters' mentalizing abilities. In addition, the regression analysis indicating that both paternal and maternal RF are significant predictors of their daughters RF suggests that each parent has a distinct and unique contribution to their daughter's RF.

### The mediating role of daughters' RF and psychological maladjustment in the relationship between parents' RF and ED severity

To the best of our knowledge, the current study is the first to empirically investigate the relationship between parents' mentalizing abilities and the severity of ED symptoms in their daughters. In line with our hypothesis, the findings demonstrate that lower levels of maternal and paternal RF contribute to an increased severity of ED symptoms among daughters. This result may be congruent with theoretical models linking deficient mentalizing abilities among patients and their parents to the use of ED symptoms as an alternative yet harmful means for dealing with psychological maladjustment [28]. Likewise, the significant relationship found between lower RF levels among parents and daughters and between poorer ED related psychological adjustment (elevated EDI-3 GPMC) among daughters seems to highlight the contribution of RF to psychological well-being in general [8, 15] and in patients with EDs in particular [27].

The regression analysis results reveal an interesting and complex picture, according to which the same mental function in mothers and fathers affect different areas of their daughters' social and emotional development. For example, we found that lower paternal (but not maternal) RF explained higher levels of social and interpersonal difficulties among the daughters, while deficient maternal (but not paternal) RF significantly explained difficulties in emotion regulation among the daughters. These findings are congruent with previous research emphasizing the important contribution of well-developed maternal RF to the development of children's emotion regulation skills [18, 71, 72] and other studies showing positive relationships between paternal (but not maternal) RF and adolescents' ability to better adapt socially [26, 70].

Interestingly, accounting for the daughters' RF has led the parents' RF variables to lose their explanatory power in relation to the EAT-26 and the EDI-3 composites, leaving the daughters' RF as the only significant explaining variable. This pattern suggests that during adolescence, the relationship between the parents' RF and ED severity and psychological maladjustment among daughters is mediated by the daughters' RF, which seems to play a more significant role than that of the parents at that age. In this context, we observed an unusual finding in the *affective problems* composition, in which adding the daughters' RF had no significant contribution beyond that of the mothers' RF. This unique result may point to the central role of maternal RF, whose contribution to the daughters' emotional regulation continues to be significant far beyond early childhood.

Finally, the mediation model provides a broad view of the relationships between the variables examined as part of the current study. The model used in this study suggests that a serial relationship exists in which mothers and fathers' low RF contribute to their daughters' low RF, which in turn is linked to higher levels of ED related psychological maladjustment, and ultimately contributes to the increased severity of ED symptoms. These results corroborate theoretical models linking deficits in parental and daughters' mentalizing ability and EDs [28].

### Limitations, advantages, and recommendations for future research

The current study has several important limitations. First, it is based on a clinical sample that included hospitalized female adolescent and young adults diagnosed with AN. This makes it difficult to generalize our findings to patients with other types of EDs, male patients, or patients with a less severe form of AN. Another limitation is the small number of participants in each group. The small sample size reflects the difficulty to recruit triads (fathers, mothers, and daughters), and this is more prominent in the AN group. Future studies using larger samples of ambulatory patients with different types of EDs may contribute to a greater statistical power that will enable to explore questions such as whether the association between parents and daughters' RF or between the parents' RF and the Daughters' ED symptomatology differ between ED patients and controls. Additionally, studies that will include males as well may answer the question whether this relation is different for male versus female adolescents. Another limitation regarding the sample is the underrepresentation of divorced families, as these families were less inclined to agree to participate in the study.

Second, we used the EAT-26 and not the Eating Disorder Examination-Question version (EDE-Q; [73]), which is more commonly used to assess the severity of ED symptoms. This was done on the premise that the EAT-26 has specific merit in the context of studying community control populations. In the current study, the HC sample did not serve merely as a control group but was also important for assessing the interactions between parental and daughters RF also in non-clinical populations. Regarding the EDI-3, it should be noted that it has better psychometric properties in the clinical compared to the non-clinical population (excellent vs. good reliability and structure validity; [52, 53].

Third, in the current study we used the AAI-RF, which is commonly used to assess parental mentalizing [74]. However, this tool assesses the general mentalizing ability and does not measure the mentalizing ability of parents regarding a specific child. Future studies using more focused tools such as the Parental Development Interview (PDI; [75]), which assesses parental mentalizing on a specific child and the parent child relationship can enrich our knowledge regarding this issue and its relation to EDs.

Finally, another limitation relates to the study's cross-sectional nature. As the participants' RF levels were assessed only at one time point, the present results cannot determine the degree to which the deficits found in parental and patients' RF represent trait or state characteristics. It is possible that the RF of patients with AN and their families, which was evaluated during inpatient treatment, was influenced by emotional overload, depression, stress and anxiety which might be increased in the patients and their families during hospitalization [76] and which can all negatively influence one's mentalizing abilities [8]. Further prospective studies are needed to examine the relationship between daughters and parents' change in mentalizing ability and the course of illness and outcomes. It is important to note that throughout the last decades efforts have been made to shift away from the view that blames parents for their child's mental difficulties. The model of mentalizing is in line with this progress, as it emphasizes the role of mentalizing as a protective factor [77].

## Conclusion and possible theoretical and clinical implications

The results of the present study provide support to theoretical models associating parental deficits in mentalizing abilities to the presence and severity of AN. This association is mediated by patients' RF and ED related psychological maladjustment. Furthermore, the results indicate that the RF of both parents continues to be associated with the daughter's RF during adolescence and young adulthood, albeit in a different manner.

Practically, these findings support previous studies suggesting that the lack of reference to emotion and internal mental states within the family might have a negative effect on the severity of an ED and its course [78–80]. Thus, our results underscore the need to improve mentalizing ability during therapy among parents and patients, and point to the merit of mentalization-based family therapy for treating AN (MBFT-ED; [81]). Additionally, the findings of this study highlight the importance of integrating fathers in the treatment of AN and the importance of focusing on their mentalizing abilities.

## Data Availability

The datasets used and/or analyzed during the current study are available from the corresponding author upon request.

## Abbreviations

ED: Eating disorder
AN: Anorexia nervosa
RF: Reflective functioning
PRF: Parental reflective functioning
ToM: Theory of mind
HC: Healthy controls
SD: Standard deviation
AN-R: Anorexia nervosa restricting type
AN-B/P: Anorexia nervosa binge/purge type
OCD: Obsessive compulsive disorder
IQ: Intelligence quotient
BMI: Body mass index
*SCID-I/P*: The Structured clinical interview for DSM-IV axis I disorders,s Patient Edition
RFS: The reflective function scale
AAI: Adult attachment interview
PDI: Parental development interview
ICC: Intraclass correlation coefficient
EAT-26: The Eating Attitudes Test-26
EDI-3: The Eating Disorder Inventory-3
EDRC: Eating Disorder Risk Composite
IC: Ineffectiveness Composite
IPC: Interpersonal Problems Composite
APC: Affective Problems Composite
OC: Overcontrol Composite
GPMC: General psychological maladjustment composite
MANOVA: Multivariate analyses of variance
ANOVA: Analyses of variance
EDE-Q: Eating Disorder Examination-Question
MBFT: Mentalization-based family therapy for eating disorders
